# Aβ inhibits SREBP-2 activation through Akt inhibition

**DOI:** 10.1194/jlr.M076703

**Published:** 2017-11-09

**Authors:** Amany Mohamed, Anissa Viveiros, Kathleen Williams, Elena Posse de Chaves

**Affiliations:** Department of Pharmacology and Neuroscience and Mental Health Institute, University of Alberta, Edmonton, Alberta, Canada

**Keywords:** Alzheimer’s disease, cholesterol/biosynthesis, cholesterol/trafficking, isoprenoids, neurons, nuclear receptors/sterol regulatory element-binding protein, prenylation, amyloid β, sterol regulatory element-binding protein-2, protein kinase B, oligomeric amyloid β_42_

## Abstract

We previously demonstrated that oligomeric amyloid β_42_ (oAβ_42_) inhibits the mevalonate pathway impairing cholesterol synthesis and protein prenylation. Enzymes of the mevalonate pathway are regulated by the transcription factor SREBP-2. Here, we show that in several neuronal types challenged with oAβ_42_, SREBP-2 activation is reduced. Moreover, SREBP-2 activation is also decreased in the brain cortex of the Alzheimer’s disease (AD) mouse model, TgCRND8, suggesting that SREBP-2 may be affected in vivo early in the disease. We demonstrate that oAβ_42_ does not affect enzymatic cleavage of SREBP-2 per se, but may impair SREBP-2 transport from the endoplasmic reticulum (ER) to the Golgi. Trafficking of SREBP-2 from the ER to the Golgi requires protein kinase B (Akt) activation. oAβ_42_ significantly reduces Akt phosphorylation and this decrease is responsible for the decline in SREBP-2 activation. Overexpression of constitutively active Akt prevents the effect of oAβ_42_ on SREBP-2 and the downstream inhibition of cholesterol synthesis and protein prenylation. Our work provides a novel mechanistic link between Aβ and the mevalonate pathway, which will impact the views on issues related to cholesterol, isoprenoids, and statins in AD. We also identify SREBP-2 as an indirect target of Akt in neurons, which may play a role in the cross-talk between AD and diabetes.

Approximately 50% of the brain’s dry mass is constituted by lipids (1). The main lipid in the brain is cholesterol. The human brain represents only 2% of the total body mass, but contains 25% of the total body cholesterol (2, 3). Therefore, it is not surprising that lipids have important functions in the brain and that dysregulation of brain lipid metabolism has been linked to brain diseases, in particular Alzheimer’s disease (AD). In the past several years, it has become clear that cholesterol homeostasis is perturbed in AD (4–6). In addition, there is increasing evidence of the importance of changes in protein prenylation in aging and in the pathophysiology of AD (6–9). Cholesterol and nonsterol isoprenoids are products of the mevalonate pathway. The mevalonate pathway is controlled by several parallel mechanisms that ensure that nonsterol isoprenoids are produced constantly without over-accumulation of potentially toxic cholesterol (6).

The mevalonate pathway is affected in AD, where there is a reciprocal regulation between cholesterol and the amyloid β (Aβ) peptides that accumulate in the brain [reviewed in (5, 6)]. Similar reciprocal regulatory mechanisms may exist between nonsterol isoprenoids and Aβ, such that protein prenylation determines the levels of intracellular Aβ (8, 10) and Aβ regulates protein prenylation (11). Neurons and astrocytes play different roles in cholesterol homeostasis in the brain. A shuttle system for cholesterol between astrocytes and neurons is key in brain cholesterol homeostasis (12, 13). Intercellular delivery pathways for nonsterol isoprenoids and oxysterols derived from the mevalonate pathway have also been suggested (14–16). Better understanding of the dysregulation of the mevalonate pathway during AD will provide important information of the pathophysiological mechanisms of disease and may lead to the identification of novel therapeutic targets.

We have demonstrated that oligomeric Aβ_42_ (oAβ_42_) inhibits cholesterol synthesis and protein prenylation in primary neurons (11). Our results indicated that oAβ_42_-induced inhibition of protein prenylation is due to the shortage of isoprenoids and that reduced protein prenylation causes impairment of neuronal intracellular trafficking and results in neuronal death. Administration of the isoprenoid, geranylgeranyl pyrophosphate (GGPP), to oAβ_42_-treated neurons recovers normal protein prenylation, corrects intracellular trafficking, and significantly reduces Aβ-induced neurotoxicity. Furthermore, we found that protein prenylation is reduced in the brain cortex of the AD mouse model TgCRND8 (11). Importantly, our data from cortical neurons suggested that the effects of oAβ_42_ on cholesterol and isoprenoid synthesis are due to reduction of the transcription factor, SREBP-2 (11).

SREBP-2 belongs to a family of transcription factors known to regulate cholesterol and fatty acid homeostasis. Cholesterol synthesis is preferentially regulated by SREBP-2, which increases expression of the majority of the enzymes of the mevalonate pathway (17–19). SREBP-2 is synthesized and inserted in the endoplasmic reticulum (ER) membranes as an inactive precursor (P)SREBP-2, which is retained at the ER by binding to the ER protein, SREBP cleavage-activating protein (SCAP) (20, 21). SCAP is both a sterol-sensing protein and an escort protein (22). Regulation of SREBPs involves intracellular trafficking and proteolytic cleavage, a process so-called maturation. The complex (P)SREBP-2-SCAP is transported into coat protein complex II (COPII) vesicles that bud from the ER and travel to the Golgi complex (23). At the Golgi, sequential proteolytic cleavage of (P)SREBP-2 by site-1-protease and site-2-protease releases the N-terminal/mature/nuclear SREBP-2 [(M)SREBP-2] that enters the nucleus to regulate gene transcription (20, 24–27). The main regulators of SREBP-2 maturation are cholesterol and oxysterols, which act by mechanisms involving SCAP and other ER integral membrane proteins called insulin-induced gene (Insig)-1 or Insig-2 (28–34). If cellular cholesterol and/or oxysterol levels are high, the complex SCAP/SREBP is retained at the ER (30, 31, 34–36). Conversely, under conditions of low cellular cholesterol, SCAP undergoes conformational changes that allow it to bind to the coat protein complex, COPII. COPII clusters the SCAP/SREBP complex into coated vesicles that bud from the ER and travel to the Golgi complex (32, 33). The exit of SCAP/SREBP from the ER represents a critical point in the feedback regulation of cholesterol metabolism (34, 35). Brown and colleagues have identified the activation of protein kinase B (Akt) as a mechanism that regulates trafficking of SCAP/SREBP from the ER to the Golgi and contributes to the control of the proteolytic cleavage of SREBP-2 (37, 38).

The main goal of this study is to identify the mechanism through which oAβ_42_ reduces levels and activation of SREBP-2. Our work is important in light of the crucial roles of cholesterol, isoprenoids, and other mevalonate-derived lipids in AD (6); and the more recent understanding that the roles of SREBPs extend well beyond lipid metabolism reaching crucial physiological processes, some of which are altered in AD (39, 40).

## MATERIALS AND METHODS

### Reagents

Aβ_42_ was purchased from American Peptide Company (Sunnyvale, CA). The same lot number was used throughout this study. Reverse Aβ_42_ was obtained from Alpha Diagnostic International Inc. Leibovitz L15-CO2 culture medium was from Invitrogen. Mouse NGF (2.5 S) was purchased from Alomone Laboratories Ltd. (Jerusalem, Israel). Immobilon PVDF was from Bio-Rad. ECL reagents were from Amersham Biosciences. Brefeldin A (BFA) (B5936) and LY294002 (L9908) were purchased from Sigma-Aldrich. MG132 (carbobenzoxy-L-leucyl-L-leucyl-L-leucinal) (474790) was obtained from Calbiochem. Z-VAD-FMK (carbobenzoxy-valyl-alanyl-aspartyl-[*O*-methyl]fluoromethylketone) (AB-001) was purchased from Kamiya Biomedical Company. All other reagents were from Fisher.

### Preparation of primary neurons

All procedures were approved by the University of Alberta’s Institutional Animal Care Committee in accordance with the guidelines of the Canadian Council on Animal Care.

### Culture of primary rat cortical neurons

Rat cortical neurons were prepared from newborn Harlan Sprague-Dawley rats of either sex (Health Science Lab Animal Services, University of Alberta). Brains were dissected and cortices were isolated and enzymatically digested by 1 mg/ml papain for 10 min at 37°C. DNase was added to the digestion mix in the last 5 min of incubation. After mechanical dissociation using a flame-polished Pasteur pipette, cell suspension was filtered through a cell strainer (40 μm nylon; Falcon) and neurons were plated on poly-D-lysine-coated wells at a density of 0.15 × 10^6^ cells/well in a 24-well plate (41). The culture medium used consisted of Neurobasal A medium (Gibco; 10888-022) supplemented with B27 (Gibco; 17504-044), Penstrep (Gibco; 15140), and glutamax (Gibco; 35050-061). Experiments started at day 7 in culture.

### Culture of primary rat sympathetic neurons

Sympathetic neurons were isolated from superior cervical ganglia of newborn Harlan Sprague-Dawley rats of either sex (Health Science Laboratory Animal Services, University of Alberta) by enzymatic dissociation with 1% (w/v) of collagenase and 0.1% (w/v) of trypsin followed by mechanical dissociation using a flame-polished Pasteur pipette. After filtration of the cell suspension through a cell strainer (40 μm nylon; Falcon), neurons were plated. The standard culture medium was L15-CO2 supplemented with 0.4% methylcellulose. Nonneuronal cells were eliminated with 10–15 μM cytosine arabinoside during the first 5–6 days in culture. Neurons were plated in 24-well dishes at a density of 1–2 ganglia per well in medium supplemented with 2.5% rat serum, 1 mg/ml ascorbic acid, and 50 ng/ml NGF. Experiments started on day 7 in culture.

### Culture of striatal cell lines, ST14A

Conditionally immortalized rat embryonic striatal cells, ST14A, were maintained in culture at the permissive temperature (33°C), as previously reported (42). Their growth medium was composed of DMEM (Gibco; 12800-017) supplemented with 10% FBS (Gibco; 12483) and 1% Penstrep (Gibco; 15140). Cells were plated in 24-well dishes at a density of 0.6 × 10^5^ cells per well and cultured for 18 h before starting the indicated treatment.

### Mouse cortices

Mutant human APP KM670/671NL+V717F Tg mice (TgCRND8) maintained on a C3H/C57BL6 background (43) were kept on a 12 h light/dark cycle and bred and housed with access to food and water ad libitum. All experiments involving animals complied with Institutional and Canadian Council of Animal Care guidelines. All mice were genotyped twice by PCR analysis of tail DNA: initially at weaning (21 days) and at euthanasia. Frontal cortices from 10-week-old male wild-type and TgCRND8 mice were used for analysis of SREBP-2.

### oAβ_42_ preparation

oAβ_42_ was prepared following a published protocol (44). Aβ peptide was initially dissolved to 1 mM in hexafluoroisopropanol and separated into aliquots in sterile microcentrifuge tubes. Hexafluoroisopropanol was dried under a stream of N_2_, and the peptide film was desiccated at −20°C. At the time of preparation, the peptide was resuspended in Me_2_SO at a concentration of 5 mM. L15-CO2 medium (phenol red-free, antibiotic-free, and serum-free) was added to bring the peptide to a final concentration of 100 μM and the peptide was incubated at 4°C for 24 h. The same batch of Aβ was used throughout the study and each oAβ_42_ preparation was characterized as in previous work (44, 45) and showed to contain monomers and oligomers of Aβ, but not fibrils.

### Analysis of the effect of oAβ_42_ on SREBPs in cultured neurons

Sympathetic and cortical neurons or ST14A cells were cultured as indicated above. When the treatment with oAβ started, the serum in the culture medium was discontinued in the case of sympathetic neurons and ST14A cells and the B27 supplement was replaced by the N2 supplement for cortical neurons. The duration of treatment with oAβ_42_ was 24 h for most experiments. In experiments using the proteasome inhibitor MG132, MG132 (5 μM) was added to the medium in the last 2 h of treatment. Neurons were harvested in RIPA buffer supplemented with protease inhibitor cocktail (Roche), 1 mM sodium orthovanadate, and 10 mM sodium fluoride. Protein mass in lysates was measured and equal amounts from each sample were separated by SDS-PAGE using gels at 10% containing 0.1% SDS. Transfer of proteins to PVDF membranes was performed overnight at 4°C in 25 mM Tris, 192 mM glycine, and 16% methanol buffer (pH 8.3). Membranes were blocked for 1 h in TBS and 0.1% Tween 20 (TTBS) containing 5% nonfat milk (blocking buffer) and incubated overnight in the primary antibody solution prepared in TTBS containing 5% nonfat milk. Primary antibodies for SREBP-2 (Abcam; ab-30682 at 1:500 dilution), SREBP1 (Santa Cruz Biotechnology; sc-13551, 1:500 in milk), ubiquitin (Cell Signaling; at 1:2,000), and β actin (Cell Signaling; 4967, 1:5,000 dilution) were used. Membranes were washed two times with TBS, two times with TTBS, and two times with TBS and then incubated for 1 h with the secondary antibody (1:2,000) in blocking buffer at room temperature with gentle agitation. Immunoreactivity was detected by ECL Prime Western blotting detection system (Amersham Biosciences). UN-SCAN-IT gel 5.3 software was used for quantification of the bands on immunoblots. Quantification was performed in low-exposure blots and the saturation was examined using two blots and the saturation-checking feature of the software. Results are expressed in ratio of pixels SREBP-2/actin referred to the untreated control. The ratios (M)SREBP-2/(P)SREBP-2 or (M)SREBP-2/total SREBP-2 cannot be used to express the results because (P)SREBP-2 levels depend on (M)SREBP-2 (46). C_SREBP_ is a control generated by overexpression of (M)SREBP-2 in St14A cells using a construct corresponding to the mature form of human SREBP-2 [kindly provided by Dr. Sipione (Sipione et al., unpublished observations)]. Cell transfections with (M)SREBP-2 were performed using Lipofectamine LTX and Plus reagent (Invitrogen; 15338-100) following the manufacturer’s instructions. C_SREBP_ was used to confirm the band of (M)SREBP-2 in immunoblots. For SREBP-1, membranes were washed in sequential steps twice with TBS, twice with TTBS, and twice with TBS followed by incubation with the corresponding secondary antibody conjugated to HRP (1:2,000) in blocking buffer at room temperature with gentle agitation. Membrane immunoreactivity was detected by incubation with Clarity Western ECL substrate (Bio-Rad) and subsequent visualization using LI-COR C-DiGit Western blot scanner. Bands were quantified using its corresponding image studio software.

### Analysis of SREBP-2 in mouse brain

Frontal cortices from 10-week-old wild-type and TgCRND8 mice were cut in small pieces and homogenized in RIPA buffer supplemented with protease inhibitor cocktail (Roche), 1 mM sodium orthovanadate, and 10 mM sodium fluoride by passing through a 22 gauge needle, 15 times. After 20 min on ice the homogenate was cleared by centrifugation at 1,000 *g* for 10 min at 4°C. Protein mass was determined and SREBP-2 was examined by immunoblotting as indicated above.

### Detection of nuclear apoptosis and caspase 3 activation

Cortical neurons were treated with oAβ_42_ in the absence or presence of the general caspase inhibitor, Z-VAD-FMK, for 24 h. Apoptotic cell death was identified by nuclear staining with Hoechst 33258. Neurons were fixed with 4% paraformaldehyde in PBS for 20 min and stained with 500 ng/ml Hoechst 33258 for 10–20 min. The percentage of apoptotic neurons was estimated by counting condensed and/or fragmented nuclei versus evenly stained nuclei. Nuclei were visualized using a Nikon TE300 inverted fluorescence microscope equipped with a Nikon digital camera, DXM-1200 (Nikon Canada, Toronto, Ontario, Canada). Images were analyzed using Northern Elite V6.0 image capture and analysis software (Empix Imaging, Mississauga, Ontario, Canada). Five hundred to one thousand neurons per treatment were counted by an observer “blinded” to the neuronal treatment. Data were analyzed using the Kruskal-Wallis test with Dunn’s multiple post hoc comparison tests. In parallel, some neurons were harvested and processed for immunoblot analysis of cleaved caspase 3. Protein mass in lysates was measured and equal amounts from each sample were separated by SDS-PAGE using gels at 10% containing 0.1% SDS. Transfer of proteins to PVDF membranes was performed overnight at 4°C in 25 mM Tris, 192 mM glycine, and 16% methanol buffer (pH 8.3). Membranes were blocked for 1 h in TTBS containing 5% nonfat milk (blocking buffer) and incubated overnight in the primary antibody solution prepared in TTBS containing 5% nonfat milk. Primary antibodies for β actin (Cell Signaling; 4967, 1:5,000) and cleaved caspase 3 (Cell Signaling; 9661s, 1:1,000) were used. Membranes were washed two times with TBS, two times with TTBS, and two times with TBS and then incubated for 1 h with the secondary antibody (1:2,000) in blocking buffer at room temperature with gentle agitation. Immunoreactivity was detected by ECL Prime Western blotting detection system (Amersham Biosciences).

### Detection of Golgi by immunofluorescence

Neurons were fixed with 4% paraformaldehyde for 20 min at room temperature, permeabilized with 0.3% saponin for 15 min at room temperature, and blocked by incubation with 2% BSA for 1 h on ice. Anti-giantin (a generous gift from Dr. Hobman, University of Alberta) at a dilution of 1:300 was prepared in the same blocking solution. Incubation with the primary antibody was performed overnight at 4°C. Secondary antibodies (Alexa 594- or Alexa 488-labeled anti-rabbit) prepared in blocking buffer (1:1,000) were incubated for 2 h at room temperature. Nuclei were stained with Hoechst (Sigma) staining (final concentration 1 μg/ml). Pictures were taken using a laser scanning confocal microscope, Zeiss LSM 710, equipped with an S-Fluor 40/1.3 oil objective using appropriate filter sets and excitation wavelengths. Within an experiment, all pictures were taken with the same setting for a particular fluorophore. If required for printing purposes, brightness and contrast were adjusted using Photoshop software. All images were adjusted with the same parameters. The pictures selected are representative of at least three separate experiments performed under identical conditions.

### Overexpression of myristoylated Akt

Primary cortical neurons were infected with recombinant adenoviruses expressing hemagglutinin-tagged myristoylated Akt (myr-Akt) or empty virus obtained from CVRC Molecular Biology Core, University of Alberta. The viral particles (at MOI 100–500) were incubated with poly-L-lysine (Sigma) for 30 min at room temperature to enhance viral infection (47). Neurons were exposed to the viral particles for 24 h. The next day, the medium was changed to fresh medium and the experiments started after 48 h of protein expression. The level of Akt expression was followed by immunoblot analysis using anti-HA antibody.

### Cholesterol synthesis

Cholesterol synthesis was determined by measuring incorporation of [^3^H]acetate using methods described by de Chaves et al. (48) and modified as described herein. Cortical neurons noninfected, infected with empty virus, or infected with virus harboring myr-Akt received the treatment indicated in each case with the addition of the radioactive cholesterol precursor [^3^H]acetic acid (100 μCi/ml) for the last 2 h. The radioactive medium was removed, neurons were washed twice with cold PBS, and cellular material was harvested and sonicated. An aliquot was separated for protein determination. A second aliquot was used for lipid isolation and quantification. Lipids were isolated from the cell lysate according to the method of Folch, Lees, and Sloane-Stanley (49) with chloroform/methanol/water at a ratio of 2:1:1 (v/v). Individual lipid separation was accomplished by TLC. TLC plates were developed in the solvent system heptane/isopropyl ether/acetic acid (60:40:4, v/v) using unlabeled cholesterol as carrier. The band corresponding to authentic unesterified cholesterol was scraped from the plate and radioactivity was measured. Radioactivity was normalized to protein mass and expressed in disintegrations per minute per microgram of protein. At least four cultures per treatment were used. To combine three experiments, the results were expressed compared with the untreated neurons, which were given a value of 100%.

### Protein prenylation

Protein prenylation was examined by extraction of prenylated proteins with Triton X-114 or recombinant GDP-dissociating inhibitors (GDIs) fused to glutathione *S*-transferase (GST) (GST-GDI).

### Separation of prenylated and unprenylated Rab using Triton X-114

The proportion of prenylated and unprenylated Rab7 was examined using extraction with Triton X-114 according to Coxon et al. (50). Two-phase mixtures of Triton X-114 were formed at 37°C so that lipophilic prenylated Rab partitioned into the detergent-rich phase, whereas unprenylated Rab remained in the aqueous phase. Cortical neurons were harvested at the end of the corresponding treatments by scraping into medium and pelleted by centrifugation at 300 *g*. Cell pellets were washed in PBS, then lysed in Tris buffer containing Triton X-114 [20 mM Tris-HCl (pH 7.5), 150 mM NaCl, 1% Triton X-114, and protease inhibitor cocktail] and incubated for 20 min at 4°C. Lysates were cleared by centrifugation at 13,000 *g* for 15 min at 4°C. Protein content was determined in the clear supernatants. In all cases, equal amounts of proteins were adjusted to the same volume with Tris buffer containing Triton X-114, were loaded on top of a cushion solution [20 mM Tris-HCl (pH 7.4) containing 150 mM NaCl, 6% sucrose, 0.06% Triton X-114, and protease inhibitor cocktail], and were incubated at 37°C for 10 min. After centrifugation at 16,000 *g* for 5 min, the top aqueous clear layer was separated from the detergent-rich oil-like droplet. Both layers were boiled with SDS sample buffer and proteins were resolved by 12% SDS- PAGE. Immunoblot analysis was performed with anti-Rab7 (R8779, 1:1,000; Sigma). Notice that prenylated Rabs always run faster than unprenylated Rabs under our experimental SDS-PAGE conditions. The lower band is present in the detergent phase. Detection of GAPDH (Abcam; 9484, 1:5,000) and COX IV (Abcam; 14744, 1:1,000 in BSA) were used to confirm proper separation of hydrophobic and hydrophilic proteins. Data are presented as the density of the band isolated in the detergent phase over the total density of the sum of the detergent and aqueous phase.

### Rab-GDI capture

GDI capture was performed according to Narita et al. (51). Neuronal lysates were prepared by homogenization of cultured neurons in buffer containing 250 mM sucrose and 3 mM imidazole (pH 7.4) with 1 mM DTT and protease inhibitors by passing through a 22 gauge needle 10 times on ice, followed by centrifugation at 300 *g* for 5 min to eliminate unbroken cells and cellular debris. Protein content was measured and equal amounts of proteins from all samples were prepared. Half of the sample was diluted 5-fold with extraction buffer (30 mM HEPES, 75 mM potassium acetate, 5 mM MgCl_2_, 100 μM ATP, and 500 μM GDP) containing 10 μM GDI-GST and incubated for 20 min at 30°C. Rabs extracted by GDI were recovered by incubating with glutathione-Sepharose 4B beads, eluted by boiling with SDS-sample buffer for 10 min, and analyzed by SDS-PAGE together with the other half of the sample that represents the input. Rab7 was detected by immunoblotting with anti-Rab7 antibodies. The concentration of GST-GDI was kept low; therefore, the extraction of prenylated proteins was limited. For that reason, blots with different exposures were used to quantify the input lanes (short exposure) and the E lanes (long exposure). Ratios between total pixels of Rab7 in GDI-bound (E)/total pixels of Rab7 in lysate before extraction (I) were calculated. Densitometric analysis of I included both bands. Data are presented as percentage of the corresponding control (untreated neurons).

### Immunoblot analysis of Akt and Sec24D

Proteins were separated by SDS-PAGE using gels at 10% containing 0.1% SDS. Transfer of proteins to PVDF membranes was performed overnight at 4°C in 25 mM Tris, 192 mM glycine, and 16% methanol buffer (pH 8.3). Membranes were blocked for 1 h in TTBS containing 5% nonfat milk (blocking buffer) and incubated overnight in the primary antibody solution prepared in TTBS containing 5% nonfat milk; except for the pAkt antibody, which was diluted in TTBS containing 5% BSA. Primary antibodies for β actin (Cell Signaling; 4967, 1:5,000), pAkt (Cell Signaling; 4058S, 1:1,000), total Akt (New England Biolabs; 1:1,000), p-p70S6 kinase (Cell Signaling; 9205, 1:1,000), p70S6K (Cell Signaling; 2708, 1:1,000), and Sec24D (Abnova; H00009871-A01, 1:500) were used. Membranes were washed two times with TBS, two times with TTBS, and two times with TBS and then incubated for 1 h with the secondary antibody (1:2,000) in blocking buffer at room temperature with gentle agitation. For Akt, pAkt, and Sec24D, immunoreactivity was detected by ECL Prime Western blotting detection system (Amersham Biosciences). UN-SCAN-IT gel 5.3 software was used for quantification of the bands on immunoblots. For p-p70S6k and p70S6K, membrane immunoreactivity was detected by incubation with Clarity Western ECL substrate (Bio-Rad) and subsequent visualization using LI-COR C-DiGit Western blot scanner. Bands were quantified using its corresponding image studio software. In all cases, immunoblot images depicted in the figures may not represent the ones used for quantification.

### RNA extraction and real-time PCR analysis of gene expression

Cultured cortical neurons were harvested in sterile 1× PBS solution containing sodium chloride, potassium chloride, potassium dihydrogen phosphate, and sodium dihydrogen phosphate (pH 7.2). Total RNA was extracted using an RNeasy kit (Qiagen) according to the manufacturer’s instructions. All samples were subjected to in-column treatment with DNaseI (Qiagen) to eliminate genomic DNA contamination. One microgram of total RNA was reverse transcribed using Superscript II reverse transcriptase (Invitrogen) and oligo-d(T) primer, and the resulting cDNAs were amplified using Power SYBR Green PCR Master Mix (Applied Biosystems) following the manufacturers’ instructions. Gene-specific primers were designed using Primer Express 3.0 software (Applied Biosystems). Primer sequences are listed in **Table 1**. Quantitative PCR analysis was performed on a StepOnePlus instrument (Applied Biosystems) by comparison with a standard curve generated by cDNA serial dilutions. The level of each mRNA was normalized to that of GAPDH. PCR cycling parameters were as follows: 50°C for 2 min, 95°C for 5 min, followed by 40 cycles of 95°C for 15 s, 60°C for 1 min, and 72°C for 40 s.

**TABLE 1.** Genes analyzed by quantitative PCR and sequences of primers used in the analysis

Gene Bank Accession Number	Gene Name	Rat Sequence
NM_001080148.1	Dhcr24	Forward: 5′-TGGAAGGAGCAGGGCAGTAA-3′
		Reverse: 5′-TCACCTGACCCATAGACACCAA-3′
NM_017008.4	Gapdh	Forward: 5′-ACTCCCATTCTTCCACCTTTG-3′
		Reverse: 5′-GGCCTCTCTCTTGCTCTCAGT-3′

### Statistical analysis

Prism 4 GraphPad computer software was used. Data are from three to five experiments with three to five replicates each. Values are expressed as mean ± SE. Statistical significance was analyzed using one-way ANOVA-Kruskal-Wallis statistic with Dunn’s multiple comparison test and is indicated by *P < 0.05, **P < 0.01, or ***P < 0.001. In experiments with only two groups, *t*-test was used.

## RESULTS

### oAβ_42_ inhibits SREBP-2 activation independently of induction of apoptosis and SREBP-2 degradation

**Fig. 1.**
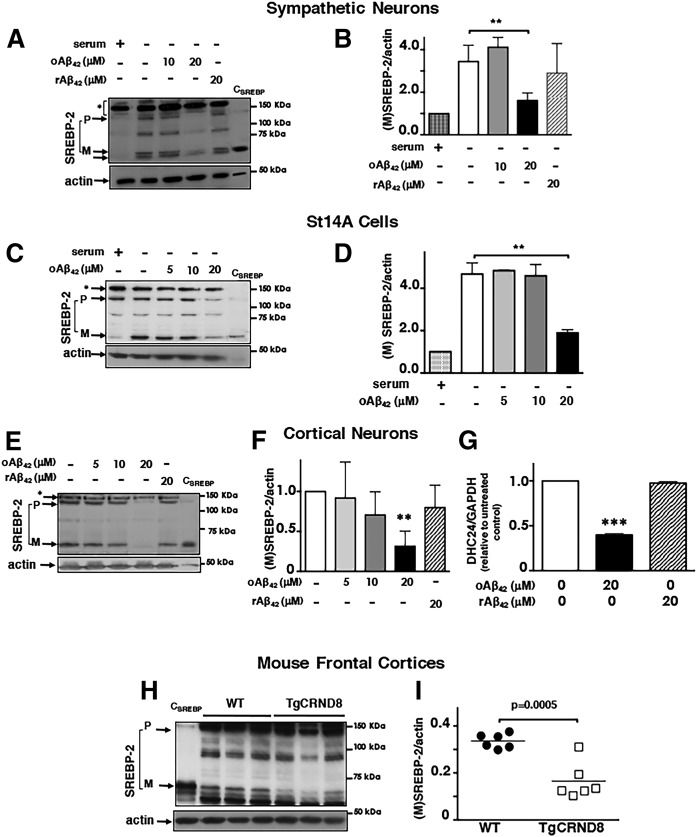
oAβ_42_ reduces neuronal SREBP-2 activation. A–G: Neurons were incubated for 24 h with different concentrations of oAβ_42_ or reverse peptide (rAβ_42_), as indicated at the top of the blots, at which time they were harvested and lysed. H, I: Frontal cortices of 10-week-old wild-type mice and TgCRND8 mice were collected and homogenized and an equal amount of protein from each homogenate was analyzed. A, C, E, H: Proteins were separated by SDS-PAGE and SREBP-2 was detected by immunoblot analysis. C_SREBP_ is a control generated by overexpression of human-(M)SREBP-2 in St14A cells and used to confirm the band of (M)SREBP-2 in immunoblots. B, D, F, I: Representations of densitometric analysis of mature forms of SREBP-2 combining at least five experiments and normalized to neurons grown in the presence of serum (B, D) or to neurons cultured in the absence of Aβ (F). G: Analysis of gene expression by real-time PCR. Data are expressed as mean ± SEM. For all experiments, **P < 0.01 and ***P < 0.005 one-way ANOVA.

SREBP-2 expression and activation are regulated by cellular sterols (20, 46), such that sterol depletion (e.g., by serum deprivation) induces transcriptional upregulation and proteolytic activation of SREBP-2. We observed that oAβ_42_ (20 μM) significantly prevented upregulation and activation of SREBP-2 induced by serum deprivation in sympathetic neurons (**Fig. 1A, B**) and in the striatal cell line, ST14A (Fig. 1C, D). oAβ also reduced SREBP-2 levels and activation in primary cortical neurons (Fig. 1E, F), a neuronal type affected in AD. The effect of oAβ_42_ on SREBP-2 was specific because the reverse peptide, rAβ_42-1_, did not alter SREBP-2 levels or activation (Fig. 1A, B, E, F). Moreover, oAβ_42_, but not the reverse peptide, rAβ_42-1_, caused a significant reduction of gene expression of the mevalonate pathway enzyme, 3β-hydroxysterol Δ24-reductase (DHCR24) (Fig. 1G), which is under the control of SREBP-2 (18, 52). Important for AD, frontal cortices of the TgCRND8 mouse, a model for AD (43), had lower levels of mature SREBP-2 compared with frontal cortices of age-matched wild-type mice at an early stage of disease progression (Fig. 1H, I).

**Fig. 2.**
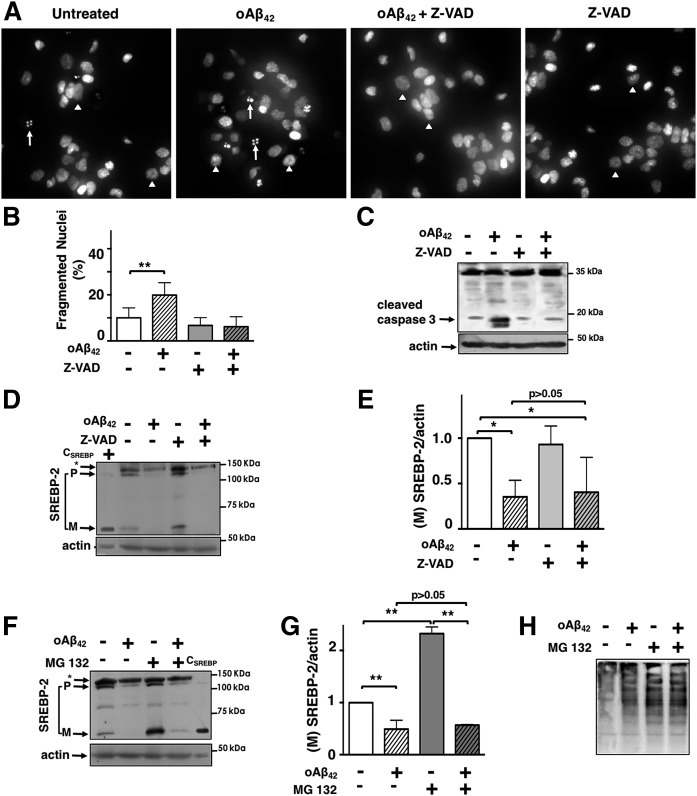
oAβ_42_ reduces (M)SREBP-2 independently of apoptosis or SREBP-2 degradation. A–E: Cortical neurons were challenged with oAβ (20 μM), ZVAD (50 μM), or a combination of both for 24 h. A: Nuclear fragmentation was evaluated by Hoechst 33258 staining using fluorescence microscopy. White arrowheads point to normal nuclei and white arrows indicate fragmented nuclei. B: Data are expressed as mean ± SEM of three experiments. Each experiment was performed in quintuplicate and 500–1,000 neurons per treatment were counted. Statistically significant differences from cultures given no Aβ (*P* < 0.01) are indicated by the symbol ** and were evaluated by the Kruskal-Wallis test with Dunn’s multiple post hoc comparison test. C: Proteins were separated by SDS-PAGE and cleaved caspase 3 was detected by immunoblot analysis. D: Proteins were separated by SDS-PAGE and SREBP-2 was detected by immunoblot analysis. C_SREBP_ is a control generated by overexpression of human-(M)SREBP-2 in St14A cells and used to confirm the band of (M)SREBP-2 in immunoblots. E: Representation of densitometric analysis of mature forms of SREBP-2 combining at least five experiments. Values have been normalized to neurons that have not received oAβ_42_ or Z-VAD. F–H: Cortical neurons were treated with oAβ_42_ (20 μM) for 24 h in the absence or presence of the proteasome inhibitor, MG132 (5 μM), for the last 2 h of treatment. F: Proteins were separated by SDS-PAGE and SREBP-2 was detected by immunoblot analysis. G: Representation of densitometric analysis of mature forms of SREBP-2 combining at least five experiments. Values have been normalized to neurons that have not received oAβ_42_ or MG132. H: Detection of ubiquitinated proteins with anti-ubiquitin antibody. Data in E and G are expressed as mean ± SEM *P < 0.05, **P < 0.01 one-way ANOVA.

To identify the mechanism by which oAβ_42_ inhibits SREBP-2 cleavage, we first excluded the possibility that the decrease of (M)SREBP-2 was due to the modest activation of apoptosis (∼20%) that takes place during the 24 h treatment with oAβ_42_ (**Fig. 2A–E**). We used the caspase inhibitor, Z-VAD-FMK, which effectively decreased oAβ_42_ -induced caspase 3 activation and reduced oAβ_42_-induced nuclear fragmentation (Fig. 2A–C). oAβ_42_ was able to decrease (M)SREBP-2 both in the absence and presence of Z-VAD (Fig. 2D, E), demonstrating that the reduction of (M)SREBP-2 was not due to neuronal apoptosis. In addition to the two-step proteolytic cleavage of the (P)SREBP-2, cellular levels of (M)SREBP-2 are also regulated by proteasomal degradation of (M)SREBP-2 (53–55). To determine whether increased proteasomal degradation of (M)SREBP-2 was responsible for its reduction in oAβ_42_-treated neurons, we treated cortical neurons with or without oAβ_42_ and/or the proteasomal inhibitor, MG132. As expected, when added individually, MG132 increased and oAβ_42_ decreased (M)SREBP, respectively (Fig. 2F, G). When added together, oAβ_42_ was still able to decrease (M)SREBP-2 and MG132 was unable to cause (M)SREBP-2 accumulation. This result suggested that the primary effect of oAβ_42_ was in the formation and not the degradation of (M)SREBP-2 by the proteasome. Importantly, oAβ_42_ did not affect the inhibition of the proteasome by MG132, as indicated by the similar increase of ubiquitinated proteins observed in neurons treated with MG132 alone and with oAβ_42_ (Fig. 2H).

### oAβ_42_ does not affect the enzymatic proteolysis of SREBP-2

**Fig. 3.**
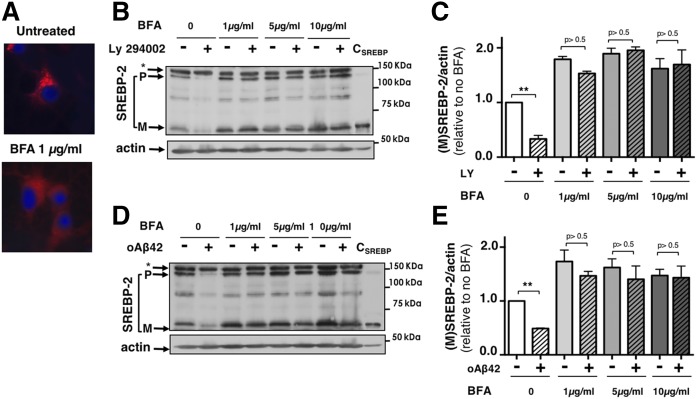
oAβ_42_ does not affect SREBP-2 proteolytic cleavage at the Golgi. A: BFA causes disassembly of the Golgi complex in cortical neurons. Notice the dense perinuclear Golgi staining in untreated neurons compared with the diffused red fluorescence in neurons that have received 1 μg/ml BFA for 5 h. B–E: BFA prevents inhibition of SREBP-2 cleavage by the PI3K inhibitor, LY294002, and by oAβ_42_. Cortical neurons were incubated with LY294002 (20 μM) (B, C) or oAβ_42_ (20 μM) (D, E) for 24 h. In the last 5 h, BFA was added to the medium at different final concentrations (1, 5, and 10 μg/ml). Neurons were harvested and lysed. Proteins were separated by SDS-PAGE and SREBP-2 was detected by immunoblot analysis. C_SREBP_ is a control generated by overexpression of human-(M)SREBP-2 in St14A cells and used to confirm the band of (M)SREBP-2 in immunoblots. C, E: The densitometric analysis of SREBP-2 cleavage combining four and five experiments, respectively. Data are expressed as mean ± SE. **P < 0.01 one-way ANOVA compared with untreated neurons.

The physiological mechanism of SREBPs’ maturation warrants that, unless required by the cell, SREBPs present in the ER remain separated from the enzymes that cleave them, which reside in the Golgi. In order to determine whether oAβ_42_ affects the enzymatic proteolysis of SREBP-2 “per se,” we used BFA, a fungal metabolite that causes redistribution of Golgi components to the ER, relocating active site-1-protease to the ER, and allowing cleavage of (P)SREPB-2 without the requirement of transport of the complex, SCAP/SREBP-2, to the Golgi (56–58). We first confirmed the effectiveness of BFA by examining the cellular distribution of the Golgi complex in neurons treated with BFA via detection of the Golgi resident protein, giantin (**Fig. 3A**). Treatment with BFA abolished the perinuclear dense Golgi staining observed in cells that did not receive BFA, indicating the reorganization of the Golgi and ER. We next tested BFA at different concentrations in cortical neurons and confirmed the increase in SREBP-2 cleavage caused by the collapse of ER and Golgi (neurons treated with BFA only in Fig. 3B, D). In the same experiment, we used the PI3K inhibitor, LY294002, as a positive control, which inhibits SREBP-2 cleavage by inhibiting Akt activation, which, in turn, prevents transport of SCAP/SREBP-2 to the Golgi (37, 38, 54). As predicted by its mechanism of action, LY294002 inhibited SREBP-2 cleavage in cortical neurons in the absence of BFA, but not in the presence of BFA (Fig. 3B, C). oAβ_42_ behaved like LY294002 in that it effectively reduced (M)SREBP-2 in the absence, but not in the presence, of BFA (Fig. 3D, E). These results indicated that oAβ_42_ does not affect the enzymatic cleavage of SREBP-2 per se and suggested that oAβ_42_ alters a step in SREBP-2 maturation that lays upstream SREBP-2 cleavage, likely the transport of (P)SREBP-2 from the ER to the Golgi.

### Aβ inhibits SREBP-2 activation by reducing cellular levels of activated Akt

**Fig. 4.**
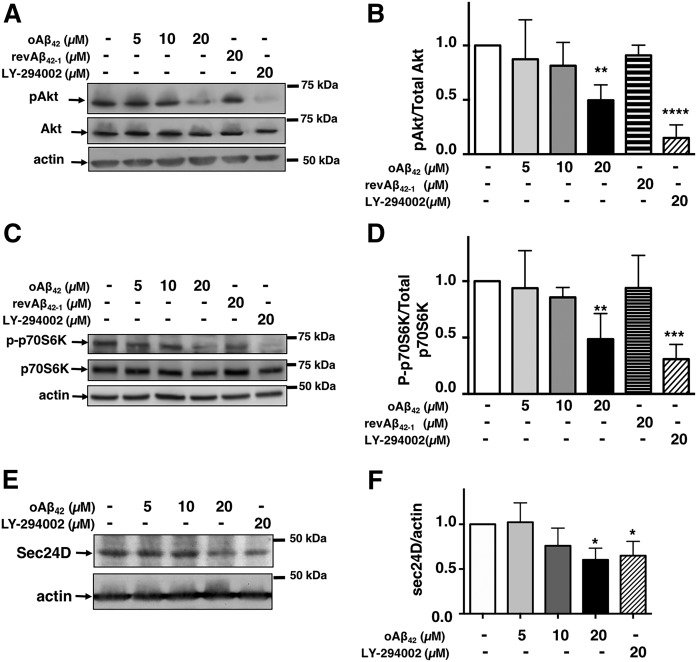
oAβ_42_ inhibits Akt signaling in cortical neurons. Cortical neurons were exposed to oAβ_42_, reverse Aβ_42-1_, or LY294002, as indicated on the top of the blots, for 24 h after which neurons were harvested and analyzed for p^Ser473^Akt and total Akt (A, B), p-p70S6K and total p70S6K (C, D), and Sec24D (E, F). Densitometric analysis (B, D, G) combines four to six experiments. Data are expressed as mean ± SEM. **P < 0.01, ****P < 0.01 one-way ANOVA with Dunnett’s multiple comparisons test.

Cholesterol and oxysterols have been recognized as the main regulators of transport of SCAP/SREBP-2 from the ER to the Golgi (28–31). In addition, Brown’s team has discovered the involvement of the PI3K/Akt pathway in this process (37, 38). Inhibition of the PI3K/Akt pathway reduces the level of (M)SREBP-2 by disrupting the transport of the SREBP-SCAP complex from the ER to the Golgi (37). Based on previous evidence indicating that Aβ may inhibit the PI3K/Akt pathway (59–61), we tested to determine whether oAβ_42_ inhibited the PI3K/Akt pathway in cortical neurons under our experimental conditions. Using immunoblot analysis with an antibody specific for the active form of Akt (p^Ser473^Akt), we examined the effect of oAβ_42_ on Akt activation. As a positive control, we used the PI3K inhibitor, LY-294002, which causes a severe reduction of pAkt in primary neurons (62). Neurons exposed to oAβ_42_ at the concentration that affects SREBP-2 (20 μM) showed significant reduction of pAkt (**Fig. 4A, B**).

Akt signals through several downstream targets, from which the mammalian target of rapamycin complex 1 (mTORC1) represents an important signaling hub. mTORC1 signals downstream to S6kinase (p70S6K). Therefore, we examined the effect of oAβ on p70S6K activation (Fig. 4C, D). As for Akt, oAβ_42_, but not the reverse peptide, Aβ_42-1_, inhibited p70S6K activation. This demonstrates that oAβ effectively inhibits the Akt signaling pathway. mTORC1 is not involved in the regulation of SREBP-2 (63), but another Akt substrate, sec24D, has been identified as one of the components of COPII vesicles that are regulated by Akt and participate in the action of Akt for the stimulation of ER-to-Golgi transport and activation of SREBP-2. Akt phosphorylates sec24 and Akt inhibitors decrease endogenous Sec24D protein levels (64). Similarly, we found that sec24D was reduced in neurons exposed to oAβ_42_ (Fig. 4E, F). This provides additional evidence that oAβ_42_ inhibits SREBP-2 activation by reducing pAkt, suggesting that this, in turn, interferes with the COPII-mediated transport from ER to Golgi.

**Fig. 5.**
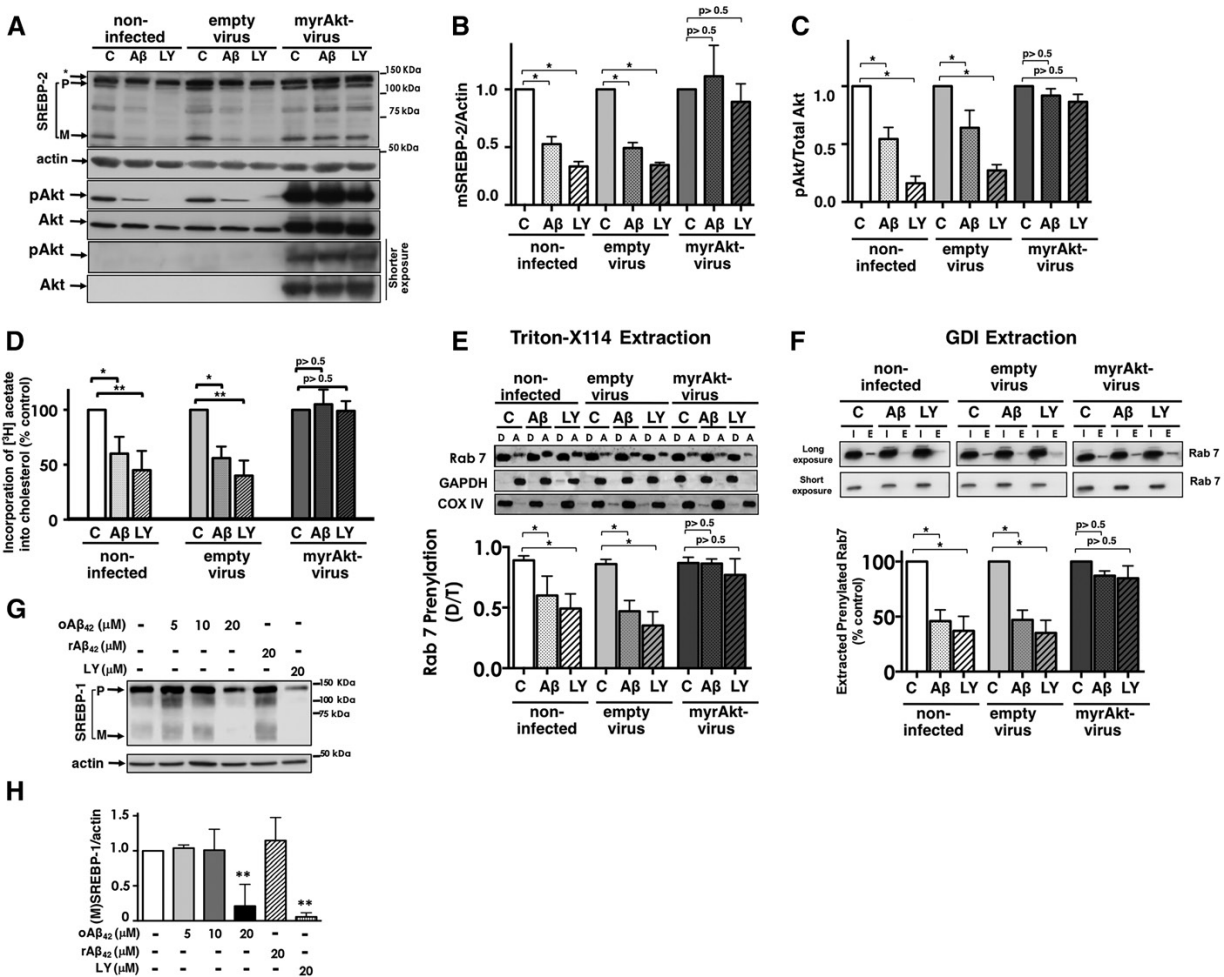
Inhibition of SREBP-2 maturation by oAβ_42_ requires inhibition of Akt. A–F: Cortical neurons uninfected or infected with an empty virus or a virus containing myr-Akt were exposed to oAβ_42_ (20 μM) or LY294002 (20 μM) for 24 h. A–C: The cells were harvested and lysed. Proteins were analyzed by SDS-PAGE. SREBP-2, pAkt, Akt, and actin were detected by immunoblot analysis. B, C: Densitometric analysis combining five experiments. Data are expressed as mean ± SEM. D: [^3^H]acetate (100 μCi/ml) was added for the last 2 h of treatment. Lipids were extracted and separated by TLC. Incorporation of [^3^H]acetate into unesterified cholesterol was calculated as disintegrations per minute and per microgram of protein and expressed as a percentage of values obtained for untreated neurons. The results are the mean ± SEM of three experiments performed in three to five replicates. E: Analysis of Rab7 prenylation by extraction with Triton X-114: neuronal lysates (equal amount of protein) were extracted with Triton X-114. Proteins in detergent (D, prenylated proteins) and aqueous (A, unprenylated proteins) phases were analyzed by SDS-PAGE and immunoblotting. Ratios between total pixels of Rab7 in detergent/total pixels of Rab 7 in detergent plus aqueous were calculated. The data are presented as percentage of control (untreated neurons in each group). Data are expressed as the mean ± SEM of three experiments. F: Analysis of Rab prenylation by Rab-GDI capture. Neuronal lysates were prepared. Half of the sample was used to assess total protein input (I). The other half was used to extract prenylated proteins with recombinant GST-GDI (E). Proteins were analyzed by immunoblot analysis. Ratios between total pixels of Rab7 in GDI-bound (E)/total pixels of Rab7 in lysate before extraction (I) were calculated. Densitometric analysis of I included both bands. Data are presented as percentage of control (untreated neurons). Data are expressed as the mean ± SEM of three experiments. G, H: Neurons were incubated for 24 h with different concentrations of oAβ_42_ or reverse peptide (rAβ_42-1_), as indicated at the top of the blots, at which time they were harvested and lysed. Proteins were separated by SDS-PAGE and SREBP-1 was detected by immunoblot analysis. H: Densitometric analysis of mature forms of SREBP-1 combining three experiments and normalized to neurons cultured in the absence of Aβ. Data are expressed as mean ± SEM. For all experiments, **P < 0.01 and ***P < 0.005 one-way ANOVA.

In order to prove that the inhibition of Akt by oAβ_42_ is the cause of the decrease in SREBP-2 and the consequent inhibition of the mevalonate pathway, we examined whether oAβ_42_ was able to inhibit SREBP-2 activation in primary neurons expressing a constitutively active form of Akt (myr-Akt) (65). oAβ_42_ and the inhibitor, LY-294002, significantly reduced SREBP-2 activation in noninfected cortical neurons and in cortical neurons infected with adenoviral particles carrying an empty vector, but expression of myr-Akt prevented oAβ_42_- and LY-induced inhibition of SREBP-2 cleavage (**Fig. 5A–C**). This finding indicates that oAβ_42_ reduces nuclear SREBP-2 cleavage via inhibition of Akt activation. Importantly in cells expressing a constitutively active Akt, oAβ_42_ was unable to inhibit the mevalonate pathway, as indicated by the lack of effect on cholesterol synthesis (Fig. 5D) and on protein prenylation (Fig. 5E, F). All together, these experiments indicate that the inhibition of the mevalonate pathway by oAβ_42_ that we have previously discovered (11) are due to inhibition of Akt, which in turn decreases SREBP-2 activation. Akt also regulates SREBP-1 (66). In agreement, oAβ42 reduced SREBP-1 activation as well (Fig. 5G, H).

## DISCUSSION

Several discoveries have indicated that cholesterol plays important roles in AD [reviewed in (5, 6, 67–69)]. The pools of cholesterol in the brain and in the periphery are strictly separated by the blood-brain barrier, and brain cholesterol homeostasis is regulated independently of peripheral organs (2). Cholesterol synthesis in situ in the brain is sufficient to meet the demands of the CNS during development and in adult life. This local synthesis may decrease with age (2, 3, 70, 71). Cholesterol biosynthesis involves successive enzymatic reactions collectively known as the mevalonate pathway. In addition to providing cholesterol, the mevalonate pathway is also the source of the short-chain isoprenoids, farnesyl pyrophosphate (FPP), and GGPP, used for posttranslational modification of proteins (protein prenylation). There is limited information on the status and regulation of the mevalonate pathway in AD (6). One report on HMG-CoA reductase (HMGCR) in AD found no change in the transcript levels of HMGCR (72). However, the Dhcr24 gene product, seladin-1/DHCR24, the enzyme that converts desmosterol into cholesterol, is downregulated in AD brains (73), suggesting that overall cholesterol synthesis could be inhibited. At the subcellular level, there is a reciprocal regulation between cholesterol and Aβ production. We, and others, have shown that Aβ peptides inhibit cholesterol synthesis (11, 74, 75). We demonstrated that oAβ_42_ inhibits SREBP-2 maturation in cultured neurons. Here, we show that the levels of (M)SREBP-2 are reduced in the frontal cortex of young TgCRND8 mice, suggesting that the negative regulation of SREBP-2 may also occur in vivo in AD. Our findings contradict a recent report of increased levels of SREBP-2 in a different mouse model of AD (76). It will be important to examine other mouse models of AD to identify whether the changes are model-specific.

Neurons might not be significantly affected by the Aβ-induced inhibition of cholesterol synthesis because, in adulthood, neurons might not synthesize cholesterol, but outsource it from astrocytes (77, 78). Conversely, inhibition of protein prenylation has significant consequences in neurons, which include but are not limited to neuronal death (11), microglia activation (79), and accumulation of full length-amyloid protein precursor (APP), APP fragments, and intracellular Aβ with a parallel decrease of secreted Aβ (8, 10). The role of protein prenylation in AD has emerged from studies using statins, which inhibit HMGCR, but may or may not inhibit protein prenylation [discussed in (6)]. On the other hand, Aβ acts on the mevalonate pathway through SREBP-2 and SREBP-2 regulates enzymes both upstream and downstream HMGCR, including FPP and GGPP synthases (17); thus, it is likely that the effect of Aβ on protein prenylation would be more severe than the effects of HMGCR inhibitors. Recently, it was reported that the precursor of Aβ, APP, also controls neuronal cholesterol synthesis through the SREBP pathway (80). These studies showed that APP levels inversely correlated with SREBP in mice and man and demonstrated that inhibition of the mevalonate pathway by APP impairs neuronal activity. The interaction of APP and SREBP-1 in the Golgi prevented the cleavage of (P)SREBP-1 and the translocation of SREBP-1 to the nucleus. Interestingly, in astrocytes, APP and SREBP1 did not interact and APP did not affect cholesterol biosynthesis, but expression of APP in neurons decreased both HMGCR and cholesterol 24-hydroxylase mRNA levels leading to inhibition of neuronal activity (80). Our data, on the other hand, indicate that Aβ_42_ does not affect SREBP-2 enzymatic cleavage. Under conditions in which the ER and the Golgi are forced to fuse, the cleavage of SREBP-2 is normal in the presence of oAβ_42_, suggesting that it is the transport of (P)SREBP-2 from the ER to the Golgi that is affected. The mechanism of action of oAβ_42_ on SREBP-2 regulation that we uncovered resembled the mechanism of the PI3K/Akt inhibitor, LY294002, closely (37).

Several studies have linked Akt to the regulation of SREBPs such that pharmacological inhibition of PI3K/Akt decreased (M)SREBP-1 in response to insulin, PDGFβ, and VEGF (81–83). It was also discovered that inhibition of the PI3K/Akt pathway regulates SREBP-2 by affecting the ER-to-Golgi transport (37, 38). Akt phosphorylates sec24, an essential COPII component involved in cargo selection for ER-to-Golgi trafficking (64). This mechanism could explain the enhancement and inhibition of SCAP transport from the ER to the Golgi induced by Akt activation and inhibition, respectively (37). In light of this evidence, we investigated the status of Akt activation and sec24 in neurons challenged with oAβ_42_. We found that levels of activated p^Ser473^Akt, the Akt/mTORC1 target p70SK, and sec24 are significantly reduced in oAβ_42_-treated neurons. Moreover, a decrease in Akt activation explains the inhibition of SREBP-2 cleavage by oAβ_42_ because Aβ was unable to inhibit SREBP-2 cleavage in neurons expressing constitutively active Akt. As expected, based on this mechanism of action, oAβ_42_ also inhibits SREBP-1 activation.

Our findings are in agreement with several previous studies that show a reduction of Akt activation due to Aβ (59, 61, 84–86) and a significant reduction of PI3K and Akt activity in AD brains (85, 87, 88) and in the brains of TgCRND8 mice (89). Some of the mechanisms that explain the inhibition of Akt by Aβ have been identified. Lee et al. (85) suggested that extra- and intracellular Aβ have different targets in the PI3K/Akt pathway; intraneuronal Aβ might block the interaction between PDK1 and Akt, thereby preventing Akt activation. There is also evidence that Aβ binds to the insulin receptor (IR) or to a receptor complex that includes the IR and interferes with insulin-induced IR autophosphorylation and consequently interferes with the PI3K/Akt cascade activated downstream of the IRs (86, 90, 91). Others have demonstrated that Aβ causes downregulation of IRs at the plasma membrane and redistribution of IRs to the cell bodies of neurons (91, 92). Thus, the decrease of Akt activation may be indirect, through the inhibition of IRs or other growth factor receptors. Recent studies showed that neuron-like cells exposed to Aβ display insulin resistance and a dysfunctional cholesterol synthesis pathway (93). Under these circumstances higher concentrations of insulin relieved the insulin-resistant phenotype and the dysregulated cholesterol signaling pathway by increasing Akt activation and the expression of enzymes of the mevalonate pathway. The activation of SREBP-2 was not investigated in this previous work. All the evidence discussed above and our results presented here contradict other studies that have found that Aβ peptides could activate Akt (94) and that the insulin signaling pathway is upregulated in AD brains (95). Akt phosphorylates a large number of targets (96). One Akt target that has received attention in the context of AD, in particular for the cross-talk between AD and diabetes, is GSK-3β. GSK-3β is involved in tau phosphorylation (97, 98) and is inhibited by Akt. Therefore, downregulation of insulin signaling could ultimately lead to both decreased glucose metabolism and increased tau phosphorylation through GSK-3 activation (99, 100).

Our work reports a novel consequence of Aβ-induced Akt inhibition, which is the inactivation of SREBP-2, which, in turn, causes inhibition of cholesterol synthesis and inhibition of protein prenylation. Novel functional roles for SREBPs have been revealed by genome-wide analyses (40, 101, 102). These global studies provided evidence that SREBPs play a more comprehensive role in physiology and metabolism through activation of additional key target genes that participate in several cellular processes (103). In this context, our findings here and in our previous study (11) indicate that SREBP-2 may also play a role in disease development, in particular in the cross-talk between AD and diabetes.
